# The stakeholders’ perceptions of the requirements of implementing innovative educational approaches in nursing: a qualitative content analysis study

**DOI:** 10.1186/s12912-021-00647-7

**Published:** 2021-07-20

**Authors:** Alireza Nikbakht Nasrabadi, Nooredin Mohammadi, Zahra Rooddehghan, Enayat A. Shabani, Fatemeh Bakhshi, Azam Ghorbani

**Affiliations:** 1 School of Nursing and Midwifery, Tehran University of Medical Sciences, Tohid Square, 1419733171 Tehran, Iran; 2 Nursing Care Research Center, School of Nursing and Midwifery, Iran University of Medical Sciences, Rashid Yasemi st, Valiasr St, 1996713883 Tehran, Iran; 3 Department of Foreign Languages, Tehran University of Medical Sciences (TUMS), TUMS International College, Keshavarz Blvd, 1415913311 Tehran, Iran; 4 School of Nursing and Midwifery, Shahid Sadoughi University of Medical Sciences, Safaeih, Fallahi, 8916877443 Yazd, Iran; 5 Medical Surgical Nursing Department, School of Nursing and Midwifery, Tehran University of Medical Sciences, Tohid Square, 1419733171 Tehran, Iran

**Keywords:** Education Research, Educational Techniques, Nursing, Qualitative Research

## Abstract

**Background:**

Improving the competencies of nurses requires improving educational methods through the use of novel methods in teaching and learning. We aim to explore the perceptions of stakeholders (including nursing education directors, faculty members and nursing students) of the requirements of implementing innovative educational approaches in nursing.

**Methods:**

In this qualitative descriptive study, 19 participants, including educational directors, faculty members, and undergraduate and graduate nursing students, were selected through the purposeful sampling method. Achieving the theoretical saturation in extracted categories was considered as a criterion for determining the sample size and the completion of sampling. The data were collected from December 2019 to May 2020 in nursing schools of Tehran, Iran, through in-depth semi-structured individual face-to-face interviews and were then analyzed based on the Graneheim and Lundman method.

**Results:**

Using qualitative content analysis, eight sub-themes and three themes were extracted. The extracted themes were ‘novel educational policymaking’, ‘Innovative education-oriented platform’, and ‘managing barriers of innovative educational approaches’.

**Conclusions:**

Developing and implementing innovative educational approaches entail providing appropriate context, structure, and required facilities by the policymaking system and educational authorities. In addition, developing capacity and related competencies of faculty members and students as the major stakeholders in employing these approaches is crucial.

**Supplementary Information:**

The online version contains supplementary material available at 10.1186/s12912-021-00647-7.

## Background

Traditional nursing educational program needs a reform to adapt with the complex nature of healthcare system as well as the evolving healthcare needs among the communities [1]. Educational programs must focus on advancing important competencies among undergraduate and graduate nursing students. These competencies might include leadership, healthcare policymaking, teamwork, collaborative and interdisciplinary care, providing community-based care, and culturally oriented care [2, 3].

Over the past decades, advances in educational science can help to adapt nursing education programs to the evolving needs of the communities [4]. These advancements, followed by the development of educational technologies, changes in psychological, educational, and learning theories which initiated changes in the traditional educational systems [5]. Concurrent with worldwide educational reforms, in 2003, the National League for Nursing asked the nursing faculty members and teachers to reflect on their conventional teaching methods and redesign nursing undergraduate curriculum to become evidence-based, flexible, dynamic, student-centered, collaborative, and technology-oriented. Employing innovative teaching methods consistent with current educational advancements were found to be the requisite for the reforms in the nursing education [4].

Currently, the nursing faculty members are receptive to the innovative educational approaches which direct them toward the main outcomes for nursing education [6]. The highlighted outcomes include becoming lifelong learners, evidence-based practitioners, and professional nurses [7]. Hence, nursing faculty members are required to apply innovative educational approaches to promote nursing education [8]. Applying new educational technologies in the nursing curriculums may also help nurses to provide care interdisciplinarily and inter-professionally [8–10]. Educational technologies include technology-based teaching strategies and virtual learning which provide a platform for nurses to become familiar with the application of information technology in the clinical settings [11, 12]. Technology is a powerful tool for effective teaching and deeper learning and can increase the opportunity for learners to create new learning experiences [13].

Short lectures, simulations, role-plays, problem-based learning (PBL), inquiry-based method, and computer-based learning are some of the innovative and creative teaching and learning methods [14]. These methods testify to the rapid growth of technology involved in learning facilitation [14, 15]. The use of technology-based education and various learning computer programs has facilitated the effective use of technology in Iran. Being learner-centered, involving students in the learning process, and creating an e-learning experience among students can be considered as the advantages of new and technology-based educational approaches [16].

Despite the advantages of applying innovative and novel educational strategies in the nursing education, there are some challenges on implementing them in the nursing curriculum. To apply innovative educational approaches, it is necessary to identify the requirements to facilitate their application, and address barriers and challenges thereof [17–20]. For teachers as facilitators, there is literature to suggest some characteristics and required competencies, such as being open to new ideas, motivated, committed, and capable to communicate with students. One critical barrier suggested is the teachers’ negative attitude toward innovative education [20]. Previous studies also mentioned some challenges associated with implementing innovative approaches in crowded classrooms, time management, and lack of supportive resources [17]. Evidence points to the current gap and the need to determine the comprehensive requirements of applying innovative approaches in the nursing educational programs.

This study contributes to the existing literatures in two ways. First, it can contribute to the limited literature on determining the requirements of implement innovative teaching approaches in the nursing educational programs. Second, our study can contribute to the literature by taking into account the perspectives of individuals who are involved in different levels of nursing education, from educational chancellors to students. In effect, we tried to provide comprehensive evidence which is applicable to a wide range of nursing education stakeholders. Hence, we aimed to understand the perceptions of stakeholders (including nursing education directors, faculty members, and nursing students) regarding the requirements of implementing innovative educational approaches in nursing.

## Methods

### Design

We conducted a descriptive qualitative study using individual face-to-face semi-structured interviews from December 2019 to May 2020. The descriptive approach could help us to provide straightforward descriptions of the perceptions of stakeholders regarding the requirements of implementing innovative educational approaches in nursing [21]. This is not a well-investigated area, and the descriptive approach was deemed most appropriate as it recognizes the subjective nature of the problem [22].

### Sample and setting

The study population consisted of nursing education directors (vice-deans and associate deans of education, and heads of clinical education departments), faculty members, and nursing undergraduate and graduate students from the nursing schools in Tehran. The inclusion criteria were: for educational directors, relevant experience of designing and implementing innovative educational programs (such as virtualization of exams or holding some virtual courses); for teachers, experience of teaching through innovative and active approaches (such as virtual education); and for students, attending classes which implemented such innovative approaches. The participants were purposefully selected. To invite teachers, we asked students to name teachers who held their classes or internships through an innovative and creative approach. To approach the directors, we asked faculty members to introduce managers who were open to apply novel programs. After each interview, the participants were asked to identify the individuals who they thought would meet the inclusion criteria. Sampling continued until non-emergence of a new concept and repetition of data in the interview in each of the three groups of participants (directors, teachers, and students) as well as achieving data and categories saturation.

### Data collection

We conducted semi-structured interviews with the participants. A.G., who had sufficient experience with conducting interviews, interviewed the participants face to face and individually based on the interview guide. In keeping with assigning the participants to three groups (educational directors, teachers and students), an interview guide was developed with different questions based on experts’ opinions and a review of the related literature. The interviews consisted of general and open-ended questions. For example, some of the managers’ questions were as follows: “Elaborate on your experience of designing and executing an instructional program using the innovative approach.”, “What limitations and challenges did you face with regard to implementing an educational program using the innovative approach?”, “What strategies did you use to manage the limitations and challenges?”. Some questions for teachers were as follows: “Explain the innovative approaches you used.”, “What facilities did you use to apply innovative approaches?”. Here are some of the questions for students: “Describe your experience of attending a class which used modern teaching methods.” (see Additional file 1).

To obtain richer descriptions, directive questions were used, such as “Can you explain more?”, or “Can you provide more examples?”. The interviews followed based on the participants’ answers. The interviews began by briefly introducing the research aim and method, after which written informed consent was obtained individually from the participants who were assured the audio-taped interviews would be kept confidential. Then the main and exploratory questions were asked [23]. The interviews lasted between 45 and 90 min, 65 min on average. Concurrent with interview data gathering, A.G. wrote relevant field notes. A.G. has participated in qualitative methods workshops and is experienced in conducting interviews. We arranged interviews based on interviewee’s convenience.

### Data analysis

In this study, the conventional qualitative content analysis approach and the Granheim and Lundman method were used to analyze the data [24]. Initially, the interviews were transcribed verbatim. Then the texts were entered into MAXQDA 10 software. The process of data analysis, coding, and the extraction of categories and themes was conducted through this software. The Graneheim and Lundman method consisted of 5 steps: First, the interviews were transcribed. Second, the interview transcripts were reviewed several times to obtain a sense of the whole. Third, based on the study objectives, the significant statements (meaning units) that were related to the participants’ understandings of the requirements of implementing innovative educational approaches were identified and each meaning unit was given an appropriate code (Table 1). To ensure coding agreement and address inter-rater reliability [25], two of the authors (A.G. and N.M.) coded the first interview transcript (about 10 pages) independently and concurrently. There was 85 % agreement between the two coders. Then, they met and resolved minor disagreements after discussion. Fourth, the codes were grouped into subcategories according to their conceptual similarities and differences, and the sub-categories were extracted. Fifth, subcategories were compared with each other for several times thereupon the latent content was identified and three themes were extracted (Table 3). The final sub-themes and themes were examined by all the authors to ensure a clear difference between categories and the fit for the data within each category.

**Table 1:** Examples of meaning units, condensed meaning units, and codes

Meaning unit	Condensed meaning unit	Code
*“In the syllabus, for instance, it is specified what lesson is to be taught for each session. For example, I say, guys, you can look at the lesson plan and say which lesson we are going to study, for instance, lesson 15 should be taught earlier or later, as long as the lessons are not related and prerequisites. A good condition is created; the student thinks he/she is involved in choosing the topic!”*	Giving students the right to choose the sequence and subject of the lesson	Student involvement in the classroom
*“Some people come and say that ‘I was your student many years ago!’, then, I ask what did you learn from me? They say, ‘I do not remember much, but I remember your class was very different, we laughed, we were happy’. Many of them say that ‘your class was a kind of class that we were satisfied with, and we were not tired!’*	Preventing fatigue and exhaustion by creating a happy and energetic atmosphere in the classroom	Creation of an energetic atmosphere in the classroom
*“We were grouped for orthopedics and a part of class was a group discussion. We had to read every session and be prepared. If we didn’t study, teachers reduced our evaluation score.”*	Pre-studying and student readiness for class	The need for student responsibility

### Trustworthiness

Considerations of rigor and trustworthiness were addressed by adopting Guba and Lincoln’s (1985) guidelines [26]. Credibility was established using member and peer-checking, reviewers’ prolonged engagement with the data, and maximum variance in participant selection. Regarding member-checking, we provided a brief report of the findings to the participants. Then, we asked them to review the data and indicate the extent to which the analyzed data reflected their experiences and perspectives. For peer-checking, the inter-rater agreement between reviewers was calculated and approved. Multiple researchers with considerable experience in qualitative research methods reflected on the analyzed data to meet researcher triangulation requirements. We considered transferability through providing a rich description of data collection, analysis processes, and findings. This method enhances the applicability and generalizability of the results to other similar contexts.

## Results

Nineteen individuals participated in this study. The participants’ demographic information is shown in Table 2. 52 % of the participants were male, 31.58 % were students from different educational grades, 47.37 % were teachers and 21.05 % were educational directors. The average teaching experience was 14.69 ± 9.51 years and the average management experience was 10.33 ± 6.80 years. The analysis of the data revealed three major themes, eight subthemes, and 936 codes (Table 3). The three major extracted themes are: ‘novel educational policymaking’, ‘innovative education-oriented platform’, and ‘managing barriers of innovative educational approaches’.

**Table 2:** The demographic information of the participants

Participant Code	position	Sex	Years of Teaching Experience (years)	Years of Executive Experience (years)	Level of Study	Semester (of studies)
D1	Educational Director	F	21	10	-	-
D2	Educational Director	F	31	15	-	-
D3	Educational Director	M	26	20	-	-
D4	Educational Director	M	11	11	-	-
T1	Teacher	F	4	-	-	-
T2	Teacher	M	25	5	-	-
T3	Teacher	M	20	-	-	-
T4	Teacher	M	20	1	-	-
T5	Teacher	F	7	-	-	-
T6	Teacher	M	5	-	-	-
T7	Teacher	F	7	-	-	-
T8	Teacher	M	11	-	-	-
T9	Teacher	F	3	-	-	-
S1	Student	F	-	-	Ph.D. Candidate	7th
S2	Student	F	-	-	Ph.D. Candidate	7th
S3	Student	M	-	-	Ph.D. Candidate	3rd
S4	Student	M	-	-	B.Sc. Student	3rd
S5	Student	F	-	-	B.Sc. Student	3rd
S6	Student	M	-	-	B.Sc. Student	3rd

*M* male, *F* female, *B.Sc.* Bachelor of Science, *S* student, *T* teacher, *D* educational director

**Table 3:** Extracted themes and sub-themes from content analysis of the data

Themes	Sub-themes	Sample codes
Novel educational policymaking	Focusing on upstream policies	The leading role of policies, creating opportunities by policies, the need for positive attitude of change-oriented policies, creating support through policies
	Stakeholder engagement	Attracting the support of managers, the need for colleagues’ engagement, clarifying the program, and making clear program goals
	An integrated educational system	Step-by-step planning, coherence of programs, allocation of time for program acceptance, creativity in planning
Innovative education-oriented platform	Expanding teacher’s teaching capacities	Making teachers informed, the need to be ready for change, change in attitude, the importance of empowerment, gaining skills to apply technology
	Preparing students	Informing students about the teaching method, creating a pleasant atmosphere in the classroom, the need for the students to be aware of their responsibility, making the classroom exciting and energetic, establishing a friendly relationship with the students
	Provision of facilities	Providing sufficient and trained workforce, appropriate structure and platform, providing the required facilities, supplying financial resources
Managing barriers of innovative educational approaches	Identifying inhibiting factors	Teachers’ resistance, students’ resistance, lack of required capabilities, lack of motivation, lack of facilities, lack of financial support
	Eliminating barriers	Accurate and principled policymaking, the need for forward-looking and open policies, allocation of resources, financial support, and workforce training

### Novel educational policymaking

This extracted theme refers to the need for developing novel policies and frameworks in nursing education. These policies can enable us to approach innovative teaching. Based on the interviewees, change-oriented policies act as the initial attempt to transform the nursing educational programs to prepare and train clinically qualified and competent nurses. ‘Focusing on upstream policies’, ‘stakeholder engagement’, and ‘an integrated educational system’ are the three sub-themes under this theme.

#### Focusing on upstream policies

Since, policies significantly impact the performance of the education system, participants believed that applying innovative educational approaches need to be congruent with the legislations. This might be a kind of guarantee for receiving financial and executive support. In this regard, one of the interviewees stated:

*“I was in charge of one of the Innovation Plans in our university. This plan aimed to transform the evaluations and assessments. It was a great opportunity! We must focus on these kinds of opportunities … Since then, our exams have been administered virtually, and our schools have been equipped with the required facilities for their implementation.”* (Educational Director 2).

Another participant said:

*“We are now in a situation in which innovative programs are supported by upstream policies … One example is launching virtual academic systems which have been introduced to universities …”* (Educational Director 1).

Hence, when upstream policies take a positive approach to change with supporting new programs and ideas, the system has an opportunity for innovation.

#### Stakeholder engagement

One of the requirements of implementing new educational methods is to attract the support of managers and the participation and cooperation of faculty members and students. The chief factor to increase stakeholders’ participation is informing them of the goals of the programs, and of how to implement each program as well as how to participate. As one of the participants stated:

*“The university directors requested us [directors in the nursing school] to improve the condition of clinical nursing education … then we presented completely our new program to promote clinical education, with the aims, visions and missions … next, it was approved by the university directors.”* (Educational Director 2).

The stakeholders’ involvement engenders their positive attitudes to assume themselves as citizens of the organization, increase their perception of self-worth, and decrease their resistance to novel educational plans. In addition, considering the interests of stakeholders and allocating motivational and incentive factors lead to an increased participation and reduced resistance in them. As one of the informants stated:

*“Privileges should be considered for people who participate in the program.”* (Educational Director 1).

#### An integrated educational system

The newly implemented educational program does not interfere with the current organized educational frameworks. Instead, it can promote the existing one and the integrity must be ensured. As one of the interviewees said:

*“Currently I teach orthopedics. What do I do for critical thinking? Nothing! I am responsible to teach the orthopedics content, not critical thinking … we need to embed critical thinking in our teaching strategies and improve the current program … through considering new strategies, we must maintain the consistency.”* (Teacher 8).

Furthermore, the participants believed that to maintain the integrity, gradual and step-by-step reforms should be taken into consideration. As one of the participants said:

*“… the freshmen don’t know the previous trends … when you tell them about the Objective Structured Clinical Examination (OSCE), they can adapt easily … contrary to the seniors’ reactions … they say ‘Oh, no! OSCE!’ For the new students, it becomes an ordinary part of the educational program … after some time, faculties and students adapted to OSCE!”* (Educational Director 4).

### Innovative education-oriented platform

This theme refers to the essential requirements of replacing traditional educational approaches with innovative educational approaches. According to the participants in this study, allocation of effective content-oriented facilities is the prerequisite to reform nursing educational programs. This theme consisted of three sub-themes: ‘expanding teachers’ teaching capacities’, ‘preparing students’, and ‘the provision of facilities’, each of which will be explained below:

#### Expanding teachers’ teaching capacities

Applying new teaching methods is related to and contingent upon teachers’ competency and capability. Initially, teachers must be mentally prepared and motivated enough to apply new approaches. Then, they should have the ability and knowledge to use these methods. To manage the classroom with new teaching methods, it is necessary for teachers to have such characteristics as calmness, flexibility, inherent passion for teaching, tendency to change, and continuous improvement. One of the participants stated:

*“… the important point is the teachers’ beliefs and attitudes toward their job and being interested in it. That is, to believe in their work and what they do … they use innovative approaches for the benefit of their students and themselves … to have faith in their abilities to apply new approaches.”* (Teacher 4).

In addition, the ability to respect students’ individual differences is one of the required characteristics for teachers. Students do not react similarly to the challenges of adapting to the newly introduced educational approaches. As one of the participants said:

*“In the beginning of the semester when students introduce themselves, I asked them to write down three of their particular characteristics and then try to apply and promote them in themselves throughout the semester.”* (Teacher 7).

The participants emphasized that the capability of teaching innovative approaches is a required competency for teachers. This competency includes a wide range of skills and abilities such as using various techniques according to the available resources, properly applying the educational technology, creating an interactive and friendly environment with students, raising energy and attraction in the class, executing active learning principles such as student-centeredness, and giving feedback to students. One of the participants stated:

*“I mainly focused on employing active learning principles rather than a certain teaching method. I used techniques such as questioning, group discussions, and virtual methods; I try to combine these methods to motivate students for active participation.”* (Teacher 8).

In relation to the energetic atmosphere in the class, one of the participants stated:

*“… some students after many years from their graduation, have told me that ‘your class was the only one that I remember! We laughed, and we were happy, and didn’t get bored and tired’. It is because I try to be energetic in the class. I try to mix it with fun and humor.”* (Teacher 4).

Some strategies were suggested by the participants to expand teachers’ teaching capacities and competencies such as attending innovational teaching methods workshops, sharing experiences, developing guidelines, attending journal clubs, and preparing virtual content. In this regard, one of the participants said:

*“… when applying a PBL teaching method by an experienced faculty member, another professor can attend this class to learn this kind of teaching practice from the competent professor.”* (Teacher 7).

#### Preparing students

New teaching methods are based on creating active learning and are learner centered. Learners’ active participation is one of the main principles of this type of training. Students need to be prepared to learn through these approaches. Obtaining the required characteristics and capacities, such as accepting their responsibility as a learner, engaging in the progression of the educational programs, being a motivated learner, shifting their tendency from teacher-centeredness to student-centeredness, is essential to their readiness. As an interviewee said:

*“Instead of spending our time on transferring information and knowledge, we should teach our students how to gain knowledge. Students should learn to be self-learners.”* (Teacher 8).

#### The provision of facilities

New educational methods require appropriate context for implementation. The requirements of running new programs include, adequate financial resources and budget allocation, creating a suitable structure and educational space, providing the necessary facilities and convenience, as well as providing a sufficient number of faculty members, clinical instructors and skilled and trained workforce. As one of the participants said:

*“… the second session of the class was held in the circular conference room. As I entered the room, I saw that the students were not sitting in rows. I enjoyed this type of sitting around a table with my friends and interacting with each other.”* (Ph.D. Candidate 3).

One of the requirements for implementing innovative programs is interdisciplinary communication to exchange needed knowledge and information. Established interdisciplinary communication help to learn from experts in other fields and disciplines when using new technologies. In this connection, one of the participants said:

*“Interdisciplinary communication is definitely essential, for example in our virtual education we need content experts in the main field, some technical individuals and engineers to turn the e-content into codes, and educational designers to design all of them.”* (Educational Director 1).

### Managing barriers of innovative educational approaches

This theme hints at recognizing and eliminating the barriers and limitations to the employment of innovative educational approaches. The recognition and elimination of the barriers and limitations entail considerable time and energy. ‘Identifying the inhibiting factors’ and ‘eliminating barriers’ are the two subthemes which can be subsumed under this theme.

#### Identifying the inhibiting factors

Various deterrents prevent the use of new teaching methods. Factors preventing change and implementation of new programs in the education system include weak policymaking, poor management, resistance of human resources to change, lack of facilities, and lack of sufficient financial resources. One of the main obstacles is the resistance of teachers to new teaching methods and changes in teaching methods. The main reasons for this resistance among teachers may include being accustomed to the routine and traditional approaches, lack of interest and motivation, lack of capability to use the novel approaches, fear of change and lack of mental preparation, and the need for spending more time and energy. As one of the interviewees stated:

*“… sometimes the facilities are prepared, but the teacher is not mentally prepared … he has long been using a specific teaching method … suddenly, they ask him to change the method he is used to … so, he becomes afraid.”* (Teacher 1).

In addition to teachers, students sometimes resist using new teaching methods. Some notable factors in this relation are as follows: unwillingness toward student-centeredness, disclaimed learning responsibility, fear from more assignments than before, impaired competencies and skills regarding such approaches, and lack of support from teachers. One of the participants stated in this regard:

*“… the professors put themselves in the margin; they ask students to prepare the content … then we must present the self-studied and learned knowledge … I think this method is useless!”* (Teacher 2).

Regarding the unsuitability of the structure and lack of facilities, the participants mentioned lack of physical space and educational facilities, limited workforce resources, and time pressure. As one of the participants stated:

*“… the current teaching method isn’t always based on our preference and choice. For example, you are in front of a class of 70–80 students, and you must present the content in just 8 sessions. Of course, you can’t say that I’m going to perform discussion-based learning! Well, this is not possible in such a crowded classroom.”* (Teacher 8).

#### Eliminating barriers

Removing barriers and deterrents to the use of new educational methods requires planning at three levels of policy, organization, and workforce. At the policy level, the mentioned barriers included incorrect policymaking, lack of foresight and forward-thinking in policymaking, individual-based policies, and unstable policies. Therefore, it is necessary to maintain principled and forward-looking policies. As one of the participants stated:

*“Universities should keep away from the prevalent and common policies of the country. The people who get involved should be purely scientific. Now, at university, we are more politicized.”* (Educational Director 2).

At the organizational level, unprincipled structure of the educational system, such as limited resources and facilities, poses an obstacle to novel approaches. At this level, removing obstacles requires overcoming shortages, creating the proper setting, budget provision, as well as providing and training human resources. One of the interviewees stated in this regard:

*“… in the master’s program, the number of students is limited, it is about 6–7 students in each class … every student has at least one turn to present a part of the course content. Like for the Teaching Skills course, we were asked to teach a part with a new method. We could all present it.”* (Ph.D. Candidate 1).

At the workforce level, the participants emphasized the importance of teachers’ preparedness to change their teaching style and manner. In this regard, one of the interviewees said:

*“One of the reasons for teachers’ resistance is that they are not familiar with the advanced educational technologies. So, their knowledge and capabilities must be updated, their attitudes toward new teaching methods and technologies need some changes, and their teaching practice must improve …”* (Educational Director 1).

## Discussion

The results show that the application of new educational methods requires the provision of requirements in the policy sector, creating the appropriate context and facilities for these approaches, and removing barriers. In countries such as Iran, where policies are centralized and the Ministry of Health and Medical Education is the main policymaker of the education system for medical sciences, appropriate policymaking is very important. The educational policies should be focused on further efforts and supports to implementing novel educational methods. In addition, they have the power to allocate privileges and grants to these kinds of effort which can possibly motivate the educational workforce in innovational practice. This finding is consistent with other studies. According to Gambhir et al. [27], one major factor affecting the quality of education is the policies and procedures of the institution, organization, and government. As Bikmoradi et al. [28] suggest, educational legislations need to become more scientific, effective, and decentralized, which can promote self-reliance and autonomy among nursing faculty members and educational managers. In addition, to plan and implement novel programs, it is required to attract the support of senior managers as well as the cooperation and participation of other stakeholders of the program. In nursing schools, collaborative leadership, delegation of authority, and sharing responsibilities between educational chancellors and faculty members must be taken into consideration [29].

Nursing teachers and faculty members are key individuals in the movement toward innovative education programs. They are required to be personally and professionally competent and motivated. The findings of the study suggest some characteristics of innovative teachers including the belief in and tendency to change, inherent passion for teaching, being mentally prepared, applying active learning strategies, and being creative to recruit teaching methods. To benefit from teachers who are prepared and capable to apply and implement new teaching methods, it is necessary to plan for the promotion of their competencies. Dozier et al. [30] found that professional development among faculty members is a strong predictor of class management and students’ involvement in the educational process. Fernandez [31] stated that empowering faculty members is essential for the development of professional identity and the transformation of teachers into change agents.

Students’ positive attitudes toward their learning responsibility, collaboration in the learning process, and self-directed learning are the requirements of innovative and active educational approaches. This is in line with DeCelle [32] who suggested that nursing teachers should identify, plan, and implement strategies which lead to self-directed and collaborative learning in students. Accordingly, teachers have an important role in developing the interest and motivation in students and attracting them to participate in the class.

Recognizing and managing barriers are among the requirements of implementing a new plan. One mentioned barrier was inaccurate policymaking in medical universities. Gharun [33] suggested that inconsistent policymaking, lack of transparency and accuracy, and lack of an assessment and accreditation system can result in a failure to achieve the macro-educational policy goals. According to Tabatabae [34] and Aqatabar et al. [35], policymaking in medical education must be comprehensive, context-based, non-hierarchical, and provident with taking future horizons into account in planning. Therefore, to remove barriers stemming from inaccurate policymaking, the following are required: correction in policymaking of the education system through the adoption of open and principled policies, together with the evaluation and accreditation of policies and programs.

Another barrier mentioned in the present study was the mismanagement of educational programs which is consistent with Nurakynova et al. [36] who found that the worthless managerial structures and strategies, being focused on short-term goals, and traditional and poor managerial methods are among the major problems of managing a modern university.

Resistance to change among teachers and students, the less-organized structure of educational programs, and lack of facilities were among the other identified barriers in our study toward implementing innovative educational approaches in nursing. Stover and Holland [37] indicated that shifting from passive lecture-based learning to active collaborative-based learning might induce negative feelings among students. Hence, the causes of resistance must be identified and eliminated through training, preparation, and allocation of motivational factors.

## Conclusions

Based on the results of the study, it can be argued that for the effective implementation of the innovative educational approaches, reforming the policymaking structures is a substantial requirement. This policymaking reform calls for the positive attitude of the legislators and the directors. The execution of innovative educational approaches opens the opportunity to improve and promote the educational programs. To this end, expanding faculty members’ capacity, preparing students, and providing facilities are among the other important requirements. Poor policymaking, resistance to change, less organized structure, and lack of facilities were recognized as barriers. The educational system should try to eliminate these barriers by means of adopting and developing proper policies, providing adequate input, fostering the culture of innovative educational approaches among the stakeholders, and preparing the faculty members.

Collecting data just through in-depth semi-structured interviews can be considered as a limitation of the present study. Future studies are recommended to use different and multiple data collection methods. Another limitation of the study might be the small sample size. However, data saturation was achieved, and maximum sampling variation was constructed. Moreover, the transferability increased through providing complete explanation of data collection and analysis processes as well as the characteristics of the participants and the context. Few studies have been conducted regarding the necessity and importance of educational policymaking in Iran and the world, which signifies the demand for further studies to be conducted in this area. Also, we recommend further studies to compare different stakeholders’ perceptions (i.e., manager, faculty member, and students).

## Supplementary Information

**Additional file 1.** Interview guide.

## Data Availability

The dataset used in the current study can be made available by the corresponding author upon reasonable request.
