# Supplementary material for: The stakeholders’ perceptions of the requirements of implementing innovative educational approaches in nursing: a qualitative content analysis study
Source: BMC Nurs. 2021 Jul 20;20:131. doi: 10.1186/s12912-021-00647-7 (PMC8290587; doi:10.1186/s12912-021-00647-7)
Supplement: Supplementary file 1 — Additional file 1. Interview guide. [file 12912_2021_647_MOESM1_ESM.docx]

**Appendix 1**

**Interview Guide**

- Main questions for educational directors

- Elaborate your experience on designing and executing an instructional program using the innovative approach.

- What limitations and challenges did you face with regarding implementing an educational program using the innovative approach?”,

- What strategies did you use to remove the limitations and rise to the challenges?”

- Main questions for teachers

-explain the innovative approaches you used.

- what facilities did you use to apply innovative approaches?

- How did you involved students?

- Main questions for students

-Explain your experience of attending a class with new education methods (such as PBL, e- learning).

- Compare this experience with a traditional class.

- What benefits you can name for the applied approach?

- What were the disadvantages?
